# Prosthetic valve infective endocarditis caused by *Cutibacterium modestum*: a case report

**DOI:** 10.1093/ehjcr/ytae313

**Published:** 2024-07-02

**Authors:** Nobuaki Shikama, Yasuo Takiguchi, Masahiko Takeda, Naoto Mori, Naoki Ishio

**Affiliations:** Department of Internal Medicine, Chiba Aoba Municipal Hospital, 1273-2 Aoba-cho, Chuo-ku, Chiba City, Chiba, Japan; Department of Internal Medicine, Chiba Aoba Municipal Hospital, 1273-2 Aoba-cho, Chuo-ku, Chiba City, Chiba, Japan; Department of Internal Medicine, Chiba Aoba Municipal Hospital, 1273-2 Aoba-cho, Chuo-ku, Chiba City, Chiba, Japan; Department of Internal Medicine, Chiba Aoba Municipal Hospital, 1273-2 Aoba-cho, Chuo-ku, Chiba City, Chiba, Japan; Department of Internal Medicine, Chiba Aoba Municipal Hospital, 1273-2 Aoba-cho, Chuo-ku, Chiba City, Chiba, Japan

**Keywords:** *Cutibacterium modestum*, Prosthetic valve infective endocarditis, 16S ribosomal RNA gene sequence, Case report

## Abstract

**Background:**

While *Cutibacterium acnes* is well known for its potential to cause acne vulgaris, postsurgical infections, and other human infections, few reports have described *Cutibacterium modestum* infections. Thus, the clinical characteristics of *C. modestum* as an infectious disease are not well understood. Herein, we describe the characteristics of a case of prosthetic valve infective endocarditis caused by *C. modestum*.

**Case summary:**

An 81-year-old man was admitted to our hospital with fever, general fatigue, and appetite loss. His past medical history included aortic valve replacement surgery and coronary artery bypass grafting for aortic valve stenosis and angina pectoris. Physical examination on admission revealed a body temperature of 39.0°C, blood pressure of 97/68 mmHg, and pulse rate of 101 b.p.m. Transthoracic echocardiography showed no prosthetic valve destruction or malfunction or obvious vegetation adhesion to the prosthetic or other valves. Bacteria initially identified as *C. acnes* were detected in two sets of anaerobic blood culture bottles collected upon admission. However, as the samples required 111 and 118 h to become blood culture–positive, the possibility of contaminating bacteria was high. Transoesophageal echocardiography revealed vegetation in the artificial valve. Repeated blood culture revealed the same bacteria; thus, contamination was ruled out, and the diagnosis of infective endocarditis was confirmed. Finally, 16S ribosomal RNA gene sequencing identified the detected bacteria as *C. modestum* rather than *C. acnes*.

**Discussion:**

Including this case, only two cases of prosthetic valve infective endocarditis caused by *C. modestum* have been reported, the characteristics of which are still poorly understood.

**Learning points**

- Patients with a prosthetic valve should be careful about *Cutibacterium* infections.
- Infections with the genus *Cutibacterium* require prolonged blood cultures to become positive and can easily be mistaken for contamination.
- Differentiating *Cutibacterium modestum* from *Cutibacterium acnes* is difficult without 16S ribosomal RNA gene sequencing.

## Introduction

*Cutibacterium modestum* is one of the several species in the genus *Cutibacterium*, including *Cutibacterium acnes*, *Cutibacterium avidim*, *Cutibacterium granulosum*, and *Cutibacterium namnetense*. While *C. acnes* is well known for its potential to cause acne vulgaris, post-surgical infections, and other human infections, less is known about the other *Cutibacterium* species. Owing to the limited reports of *C. modestum* infections, the clinical characteristics of *C. modestum* as an infectious disease are not well understood. Herein, we report a case of prosthetic valve infective endocarditis caused by *C. modestum*.

## Summary figure

**Table:** 

2018		Aortic valve replacement surgery, coronary artery bypass grafting, and left atrial appendage closure for aortic valve stenosis and angina pectoris
2023	Day-30	Low-grade fever
2023	Day 0	Admission because of fever up to 39.0°C, general fatigue, and loss of appetite
2023	Day 5	Bacteria, initially identified as *C. acnes*, detected in two sets of anaerobic blood culture bottles collected at the time of admission (Day 0)
2023	Day 8	Blood culture retest
2023	Day 10	Transoesophageal echocardiogram revealed vegetation on the annulus of the artificial biological aortic valve
2023	Day 11	Antibiotic treatment [ampicillin/sulbactam (ABPC/SBT) 3 g intravenously, every 6 h]
2023	Day 13	Detection of the same bacteria in two sets of blood culture anaerobic bottles from samples taken at the time on Day 8
2023	6 weeks after admission	Switch to oral antibiotics (amoxicillin 1500 mg/day)

## Case presentation

An 81-year-old man was admitted to our hospital in May 2023 with fever, general fatigue, and appetite loss. The patient’s past medical history included aortic valve replacement surgery, coronary artery bypass grafting (CABG), and left atrial appendage closure for aortic valve stenosis and angina pectoris, in 2018. The week before admission, the patient had complained of a low-grade fever within the previous month. Owing to the worsening symptoms and hyperthermia, the patient was urgently hospitalized for further evaluation.

**Figure 1:**
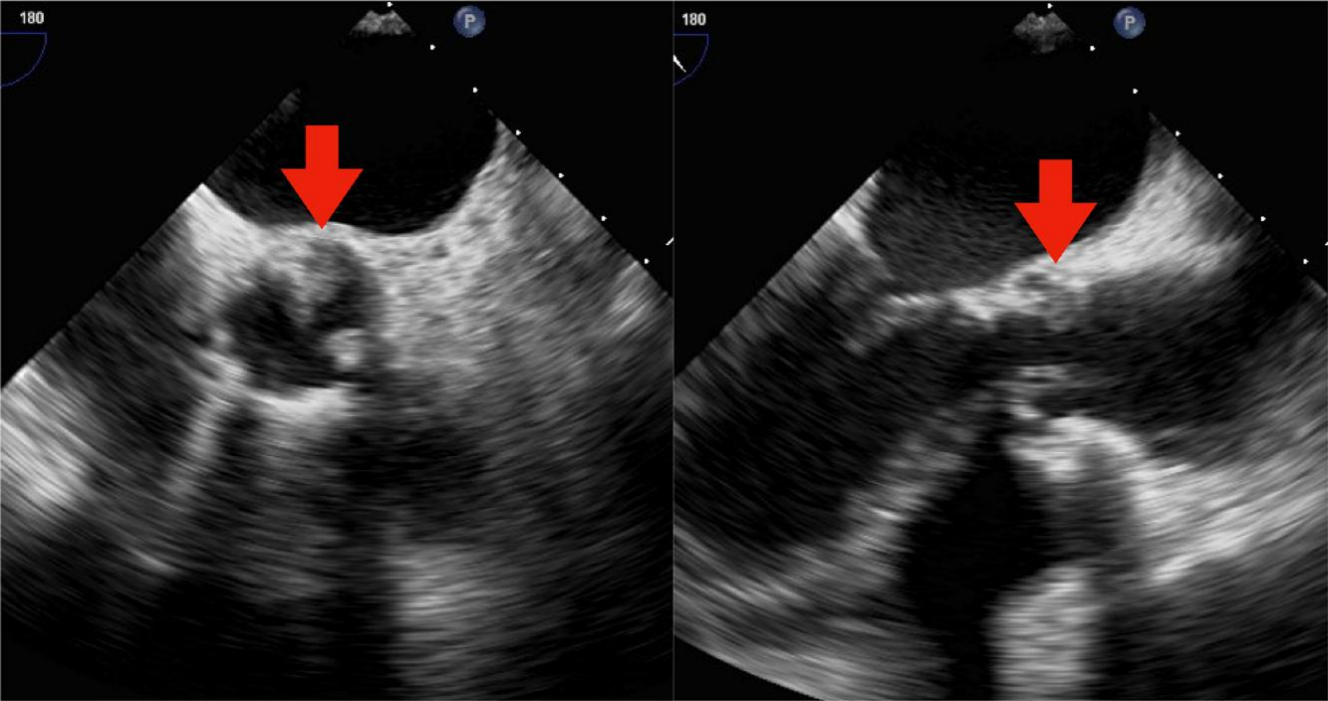
Transoesophageal echocardiography performed in May 2023 showing prosthetic aortic valve vegetation.

Physical examination on admission revealed a body temperature of 39.0°C, blood pressure of 97/68 mmHg, and pulse rate of 101 b.p.m. No murmurs, crackles, or peripheral oedema were observed. Chest radiography revealed blunting of the bilateral costophrenic angles and mild widening of the cardiothoracic ratio with no pulmonary congestion. Contrast computed tomography of the chest, abdomen, and pelvis revealed bilateral pleural effusion. However, no obvious pneumonia, abscess, or thrombosis was identified. Transthoracic echocardiography did not reveal destruction or malfunction of the prosthetic valve or obvious vegetation adhesions to the prosthetic valve or other valves. The laboratory data included a white blood cell count of 8110/mm^3^, red blood cell count of 3.39 × 106/mm^3^, haemoglobin concentration of 11.2 g/dL, haematocrit of 33.8%, platelet count of 5.7 × 104/mm^3^, and a C-reactive protein concentration of 2.3 mg/dL. The liver and renal functions were within normal ranges. The bacteria, initially identified as *C. acnes*, were detected in two sets of anaerobic blood culture bottles collected at the time of admission. However, because it took 111 and 118 h for the two sets to become blood culture–positive, respectively, the possibility of contaminating bacteria was high. Because no symptoms of collagen disease, including nephritis or arthritis, were present and no other fever source of fever could be identified, a transoesophageal echocardiogram was performed, which revealed vegetation on the annulus of the artificial biological aortic valve (*Figure 1*; Supplementary material online, *Videos S1* and *S2*). Therefore, the blood culture was repeated, which revealed the same bacteria in the two sets of anaerobic bottles; thus, contamination was ruled out, and the diagnosis of infective endocarditis was confirmed. As it is difficult to classify the *Cutibacterium* genus based solely on bacterial morphological staining, we ordered 16S ribosomal RNA (rRNA) gene sequencing from Miroku Medical Laboratory (Nagano, Japan). The sequencing results from the isolated Gram-positive bacilli showed 99.9% (1335/1336 bp–positive matches) identity with that of *C. modestum* strain M12 (NR_174227). Therefore, the bacteria detected were not *C. acnes* but rather *C. modestum*.

Thereafter, antibiotic treatment [ampicillin/sulbactam (ABPC/SBT), 3 g administered intravenously every 6 h] was continued for approximately 6 weeks, after which the inflammatory response was negative and the clinical course was uneventful. After consultation with a cardiovascular surgeon, we observed no bacterial valve destruction and considered that the infection was under control with penicillin antibiotics. Since reoperation was a high-risk procedure due to his advanced age,^1^ previous CABG, and prosthetic aortic valve endocarditis,^2^ oral antibiotics (amoxicillin 1500 mg/day) were administered for as long as possible.^3^ The plan is to continue closely monitoring the patient’s inflammatory response through blood sampling and the condition of the prosthetic valve through echocardiography at outpatient clinics.

## Discussion

*Cutibacterium* isolated from blood cultures is often considered a contaminant rather than a pathogen that causes true infections; therefore, it is difficult to diagnose as an infectious disease. However, a Swedish group that examined 312 patients with positive bacterial blood cultures found that 49 (16%) were true infections, most commonly by *C. acnes* (87%).^4^ A registry study reported a relatively low incidence of infective endocarditis caused by *C. acnes* (∼0.3%), that it is more common in men, and that it is more likely to occur in patients with prosthetic valve replacement.^5^ A recent registry study observed that 24 of 1325 patients (∼1.8%) with a diagnosis of infective endocarditis were confirmed to have *C. acnes* infection. Furthermore, 23 of these 24 cases (96%) had an artificial valve (22 patients) or an annuloplasty ring (1 case).^6^ Another registry study focusing on infective endocarditis of artificial valves reported *C. acnes* in 47 of 780 patients (6%).^7^ A comparison of registry studies reported in 2007^5^ and 2017^6^ suggests that the frequency of infective endocarditis caused by *C. acnes* is still rare but increasing. One reason for this is the increasing number of patients with prosthetic valves in countries with aging populations. As the number of patients receiving prosthetic valves increases, and as a result, the number of *C. acnes* infections is also likely to rise. Another challenge is diagnosis, as the detection of infective endocarditis due to *C. acnes* in blood culture requires culture times ≥5 days.^8^ Two other registry studies suggested a median time for blood culture positivity of 7 days.^6,9^ The longer blood culture time increases the frequency of detecting contaminating bacteria and may also be a contributing factor to the difficulty in diagnosis of infective endocarditis.

As described above, the characteristics of infective endocarditis caused by *C. acnes* have been clarified to some extent. In brief, the condition is relatively common in male patients with prosthetic valves; however, the time required for the bacteria to be detected makes diagnosis difficult and may delay treatment. Only a few case reports have described *C. modestum* as an infectious disease, including one case of suppurative spondylitis,^10^ one case of implant-related hip infection,^11^ and two cases of native vertebral osteomyelitis.^12,13^ To the best of our knowledge, only one case of infective endocarditis caused by *C. modestum* has been reported, in which glomerulonephritis developed from cryoglobulinaemia due to infective endocarditis.^14^ Although only two total cases of infective endocarditis caused by *C. modestum* have been reported, including the present case report, in both cases, aortic valve replacement surgery was performed and the patients were male. In our case, blood culture positivity required ∼5 days; thus, a long culture period is also likely to be required for *C. modestum*. Therefore, infective endocarditis due to *C. modestum* may have similar properties to that caused by *C. acnes*. In addition, standard culture tests and staining can lead to the confusion between *C. acnes* and *C. modestum*.^11^ In our case, the initial blood culture results confirmed the diagnosis of *C. acnes;* however, 16S rRNA gene sequencing revealed that the infection was caused by *C. modestum*. In previous cases diagnosed as infective endocarditis due to *C. acnes* without genetic analysis such as 16S rRNA gene sequencing, the possibility of infection by *C. modestum* cannot be ruled out. Therefore, further investigation is required to determine the relationship between *C. modestum* and infective endocarditis.

## Supplementary Material

ytae313_Supplementary_Data

## Data Availability

The data are available from the corresponding author upon reasonable request.

## Lead author biography

After graduating from Dokkyo Medical University, Dr Nobuaki Shikama trained at the Department of Cardiovascular Medicine, Chiba University Hospital. He then served as the director of the cardiovascular internal medicine department at Chiba Aoba Municipal Hospital and currently serves as the director of the regional cooperation office. In his free time, he enjoys long rides on his road bike.

## Supplementary material

**Consent**: Written informed consent was obtained from the patient for the publication of this case report in line with COPE guidance.

**Ethics approval:** Not applicable.

**Funding:** None declared.
